# Effect of low dose acetylsalicylic acid and anticoagulant on clinical outcomes in COVID‐19, analytical cross‐sectional study

**DOI:** 10.1002/hsr2.699

**Published:** 2022-07-11

**Authors:** Muhammad B. Malik, Samar A. Amer, Eric Merrell, Ronald Russo, Jeffrey B. Riley, Austin Scro, Elizabeth James, Anderson Anuforo, Soumya Adhikari, Rosalie Siciliano, Philip Chebaya, Edward Darling, Michael Kuhn, Gary Nieman, Ahmed Shawkat, Hani Aiash

**Affiliations:** ^1^ Department of Medicine SUNY Upstate Medical University Syracuse New York USA; ^2^ Department of Public Health and Community Medicine Zagazig Medical University Zagazig Egypt; ^3^ Cardiovascular Perfusion College of Health Professions SUNY Upstate Medical University Syracuse New York USA; ^4^ SUNY Upstate Medical University Syracuse New York USA; ^5^ Department of Surgery SUNY Upstate Medical University Syracuse New York USA; ^6^ Department of Pulmonary and Critical Care SUNY Upstate Medical University Syracuse New York USA; ^7^ Department of Family Medicine Suez Canal University Egypt

**Keywords:** acetylsalicylic acid, anticoagulation, ASA, COVID‐19, preventative medicine

## Abstract

**Background and aims:**

The therapeutic strategy for the treatment of known sequelae of COVID‐19 has shifted from reactive to preventative. In this study, we aim to evaluate the effects of acetylsalicylic acid (ASA), and anticoagulants on COVID‐19 related morbidity and mortality.

**Methods:**

This record‐based analytical cross‐sectional study targeted 539 COVID‐19 patients in a single United States medical center between March and December 2020. Through a random stratified sample, we recruited outpatient (*n* = 206) and inpatient (*n* = 333) cases from three management protocols, including standard care (SC) (*n* = 399), low‐dose ASA only (ASA) (*n* = 112), and anticoagulation only (AC) (*n* = 28). Collected data included demographics, comorbidities, and clinical outcomes. The primary outcome measure was inpatient admission. Exploratory secondary outcome measures included length of stay, 30‐day readmission rates, medical intensive care unit (MICU) admission, need for mechanical ventilation, the occurrence of acute respiratory distress syndrome (ARDS), bleeding events, clotting events, and mortality. The collected data were coded and analyzed using standard tests.

**Results:**

Age, mean number of comorbidities, and all individual comorbidities except for asthma, and malignancy were significantly lower in the SC compared to ASA and AC. After adjusting for age and comorbidity via binary logistic regression models, no statistical differences were found between groups for the studied outcomes. When compared to the SC group, ASA had lower 30‐day readmission rates (odds ration [OR] 0.81 95% confidence interval [CI] 0.35–1.88, *p* = 0.63), MICU admission (OR 0.63 95% CI 0.34–1.17, *p* = 0.32), ARDS (OR 0.71 95% CI 0.33–1.52, *p* = 0.38), and death (OR 0.85 95% CI 0.36–1.99, *p* = 0.71).

**Conclusion:**

Low‐dose ASA has a nonsignificant but potentially protective role in reducing the risk of COVID‐19 related morbidity and mortality. Our data suggests a trend toward reduced 30‐day readmission rates, ARDS, MICU admissions, need for mechanical ventilation, and mortality compared to the standard management protocol. Further randomized control trials are needed to establish causal effects.

**Abbreviations**
AC: anticoagulation only group
ANOVA: analysis of variance
ARDS: acute respiratory distress syndrome
ASA: acetylsalicylic acid, ASA only group
CI: confidence interval
CNS: cerebrovascular disease
COPD: chronic obstructive pulmonary disease
CVD: cardiovascular disease
DM: diabetes mellitus
HR: hazard ratio
HTNh: hypertension
IRB: institutional review board
ISTH: International Society on Thrombosis and Hemostasis
LOS: length of stay
MICU: medical intensive care unit
OR: odds ratio
SARS‐CoV‐2: severe acute respiratory distress syndrome coronavirus‐2
SC: standard care group
SD: standard deviations
SPSS: Statistical Package Software Statistics

## 1 INTRODUCTION

Coronavirus disease 2019 (COVID‐19) is a novel respiratory disease caused by the severe acute respiratory syndrome coronavirus (SARS‐CoV‐2). In the United States, there have been over 32 million cases and 580,000 deaths from the virus as of May 2021.^1^ The majority of COVID‐19 cases are mild to moderate, but 14% of cases are severe with up to 5% of cases in critical condition experiencing respiratory failure, shock, or multisystem dysfunction.^2^ In early 2020, hospitalizations due to COVID‐19 accounted for 14% of reported cases in the United States, with 2% admitted to the intensive care unit (ICU).^2^ The vast majority of COVID‐19 cases do not require hospitalization, and those patients are sent home to quarantine without further medical intervention. As a result, less information regarding complications arising from outpatient COVID‐19 cases is documented.

Current guidelines for managing outpatients with COVID‐19 include mostly supportive care. Of note, there is no current guideline recommending low‐dose acetylsalicylic acid (ASA) for prehospital COVID‐19 patient.^3^ ASA has been used in medicine for over a century and is known for its pleiotropic effects.^4^ Specifically, ASA is known to reduce platelet activation by inhibiting the formation of thromboxane A2 but has also been found to have direct antiviral properties.^4^, ^5^ Severe COVID‐19 infection is predominantly a multisystem inflammatory process resulting in disease pathogenesis manifesting as ARDS, endothelial dysfunction, and coagulopathy.^6^, ^7^, ^8^ With ASA's anti‐inflammatory, antiplatelet aggregation, and anticoagulation properties, it is often prescribed as a secondary preventative measure in cardiovascular disease.^8^, ^9^ Given this, it has been suggested that ASA and other antiplatelet agents may play a preventative role in reducing coagulopathy complications that have been shown to arise in 25%–42% of COVID‐19 patients.^10^, ^11^ Low‐dose ASA may also improve overall clinical outcomes in COVID‐19 patients by reducing ICU admission, time spent on mechanical ventilation, and hospital mortality.^8^

A recent study showed that patients with COVID‐19 who received low‐dose ASA were associated with a lower incidence of requiring mechanical ventilation, ICU admission, and in‐hospital mortality.^11^ Other studies have shown that ASA helps reduce mortality in patients with COVID‐19 who have taken ASA, but the evidence lacks high certainty.^6^ However, some studies suggest no association between the use of low‐dose ASA and mortality in COVID‐19 patients.^7^ Given the wide effects of low‐dose ASA and previous studies suggesting conflicting results of ASA administration in COVID‐19 patients, we compared the outcome of low‐dose ASA use in primary prevention of hospitalization and improving overall clinical outcomes in patients with COVID‐19.

## 2 MATERIALS AND METHODS

This record‐based analytical cross‐sectional study collected a stratified random sample of 539 patients from a total of 2714 available confirmed COVID‐19 patients at a single upstate New York, USA medical center between March 1, 2020, and December 1, 2020. Patient information was collected from both the inpatient and outpatient settings.

The criteria for inclusion into the study included all confirmed COVID‐19 cases that were identified by a positive result of real‐time polymerase‐chain‐reaction, aged 18–89 years old, and exclusion criteria included: current pregnancy, current clopidogrel use, current incarceration, a prior history of thromboembolism or hereditary hypercoagulable disorder, and surgery or hospitalization for reasons other than COVID‐19‐related illness during or 14 days before the indexing period.

### 2.1 Sample size

The sample size was estimated according to the following equation: (*n* = Z2) *P* (1 − *P*)/*d*2. From the total number of 2714 potentially relevant cases, Based on the reported cases at the study setting between March 1, 2020, and December 1, 2020, and based on the prevalence of our primary outcome (inpatient admission) in the United States, hospitalizations due to COVID‐19 accounted for 14% of reported cases,^2^ at a 95% confidence level and 80% power of the study, as a stratified sample to represent the three management protocols and to increase the probability of detecting secondary outcomes like ICU admission, which was reported to be 2% in the United States.^2^ The calculated sample size was 539. Patients who met the selection criteria were randomly recruited and stratified per proportions and distributed into the three management protocols per care setting into: standard care (SC) (*n* = 399) (74.0%), ASA only (ASA) (*n* = 112) (20.8%), and anticoagulation only (AC) (*n* = 28) (5.2%) with the associated percentage of total patients being outpatient (*n* = 206) (37.8%) and inpatient (*n* = 333) (62.2%). Patients who reached inpatient care through hospital admission were not counted in the outpatient group.

Groups were defined as:

- Patients in the SC group: those taking neither ASA nor therapeutic anticoagulation as a home medication. If admitted to the inpatient setting, these patients may have received prophylactic dosing of anticoagulants, acetaminophen, or nonsteroidal anti‐inflammatory drugs if clinically indicated. Based on hospital standards of care, all patients admitted to the hospital who met the criteria for deep vein thrombosis received pharmacologic prophylaxis through either subcutaneous enoxaparin 40 mg daily or unfractionated heparin 5000 mg three times daily. In circumstances inpatient where it was clinically indicated for therapeutic anticoagulant use, including novel oral anticoagulants, warfarin, and heparin products, the patient was not crossed over to the AC group. No other exclusion criteria were required for this group.
- Patients in the ASA group take 81–160 mg of ASA orally every day. Due to hospital standards, patients in this group were also taking prophylactic dosing of anticoagulants as in the SC group. Patients taking therapeutic anticoagulants were excluded from this group.
- Patients taking any anticoagulant agent as a home medication at therapeutic dosing: patients taking any anticoagulant agents alone at therapeutic dosing as a home medication. Anticoagulants include novel oral anticoagulants, warfarin, and heparin products. Patients taking low‐dose (81–162 mg PO daily) or taking any anticoagulation agents during the indexing period were excluded from this group.

All groups were treated with the same basic management protocol based on guidelines by Upstate University Hospital.

### 2.2 Study data

Information obtained from patient records included demographic data, comorbidities, clinical outcome data (primary and secondary as listed below in study objectives), and mortality. *Medication history* was used to identify patients taking ASA and other anticoagulants. *Inpatient admission* was defined as a hospital stay of at least 24 h. *Respiratory data* collected included the presence of acute respiratory distress syndrome (ARDS) defined by the Berlin criteria, the need for mechanical ventilation, and total intubation time until extubation or death. *Bleeding events* were defined by International Society on Thrombosis and Hemostasis criteria as clinically overt bleeding accompanied by a decrease in the Hb level of ≥2 g/dl or transfusion of ≥2 units of packed red cells, occurring at a critical site, or resulting in death. These events included gastrointestinal bleeding, intracranial bleeding, and any bleeding that required surgical or interventional radiology intervention. Clotting events included deep venous thrombosis, pulmonary embolism, and peripheral arterial occlusion occurring during the hospitalization. Mortality was defined as death occurring following the diagnosis of an acute COVID‐19 infection.

### 2.3 Study objectives

The objective of this study was to study the effects of ASA and anticoagulants on COVID‐19‐related outcomes. The primary outcome measure in this study was inpatient hospital admission. Additionally, exploratory methods were used to evaluate secondary outcomes including length of hospital stay, 30‐day readmission rates, medical intensive care unit (MICU) admission, need for mechanical ventilation, the occurrence of ARDS, bleeding events, clotting events, and mortality.

### 2.4 Statistical analysis

The collected data were coded and analyzed using Statistical Package Software Statistics (SPSS) version 26.0 (IBM Corp.). Qualitative data summarization was prepared using frequency (*f*) and percentage (%); *χ* ^2^ was used for the analysis of these variable. Mean, standard deviation (SD), two‐sided independent *t*‐test, and two‐sided analysis of variance (ANOVA) test were used for quantitative, normally distributed data summarization, and analysis. While median, Interquartile range, and two‐sided Kruskal–Wallis tests were used for summarization, and analysis of nonparametric quantitative variables. *p* value is the probability of finding the observed or more extreme results when the null hypothesis is true and considered significant when *p* < 0.05; the 95% confidence interval (CI) will contain the true parameter value. 95% of the time, a study has to be repeated many times using different samples.

Before using logistic regression, all assumptions of logistic regression were satisfied; mortality is an outcome. Multicollinearity in regression analysis occurs when two or more predictor variables are highly correlated to each other, for example, age, number of comorbidities, and variance inflation factor (VIF) values were rounded to 1.1, which means no correlation between a given predictor variable and any other predictor variables in the model (Table 1).

**Table 1:** Coefficients.

Group (0 = control; 1 = ASA; 2 = AC)	Model		Unstandardized coefficients		Standardized coefficients	*t*	Sig.	Collinearity Statistics	
			*B*	Std. error	*β*			Tolerance	VIF
0	1	(Constant)	66.617	3.864		17.241	0.000		
		BMI	−0.685	0.123	−0.353	−5.550	0.000	0.895	1.117
		Comorbidity count	4.943	0.709	0.443	6.973	0.000	0.895	1.117
1	1	(Constant)	78.339	4.252		18.424	0.000		
		BMI	−0.543	0.141	−0.417	−3.864	0.000	0.865	1.157
		Comorbidity count	1.396	0.662	0.227	2.108	0.038	0.865	1.157

Abbreviations: AC, anticoagulation only group; ASA, acetylsalicylic acid; BMI, body mass index.

The following tests were used to predict COVID‐19 outcomes, including primary outcomes (inpatient admissions) and exploratory secondary outcomes (30‐day readmission rates, the occurrence of ARDS, ICU admission, and death) through the following tests: Hosmer and Lemeshhow test, *χ* ^2^, degree of freedom (*df*), Omnibus test model *χ* ^2^, Cox and Snell *R* ^2^, Nagelkerke *R* ^2^, and overall percentage.

## 3 RESULTS

There was an observed statistically significant difference in demographics (age, body‐mass index) and comorbidities (cardiovascular disease, cerebrovascular disease, chronic obstructive pulmonary disease [COPD], obstructive sleep apnea [OSA], hypertension, diabetes mellitus, and dyslipidemia) and total number of comorbidities between the SC group and other groups (ASA and AC). Patients on ASA, and AC were significantly older, had higher body mass index's (BMI), and a greater number of comorbidities than patients in the SC group (*p* < 0.001). There was no statistically significant difference between sex, asthma, or malignancy. Diabetes mellitus (*n* = 35) (47.3%), and dyslipidemia (*n* = 78) (69.6%) were the most frequent comorbidities observed among patients in the ASA group. While cardiovascular disease *n* = 19 (67.0%), OSA (*n* = 10) (35.7%), and COPD (*n* = 9) (32.1%) were most frequent among patients in the AC group (Table 2).

**Table 2:** The demographic and clinical characteristics of the studied groups.

	All the studied groups				Outpatients only (*n* = 206)			
Groups	SC group, no = 399, F (%)	ASA group, no = 112, F (%)	AC group, no = 28, F (%)	*p*	SC group, no = 176, F (%)	ASA group, no = 25, F (%)	AC group, no = 5, F (%)	*p*
Sex				**>0.05**				
Female	214 (53.6)	52 (46.4)	12 (32.9)		110 (62.5)	16 (64.0)	3 (60.0)	**>0.05**
Male	185 (46.4)	60 (53.2)	16 (57.1)		66 (37.5)	9 (36.0)	2 (40.0)	
Age (year) Mean ± SD	(48.8 ± 18.9)a	(65.9 ± 10.9)b	(66.8 ± 18.4)b	<0.001*	38.5 ± 13.9	63.1 ± 6.5	60.2 ± 22.3	<0.001*
Age groups (years)								
<54	238 (59.6)	15 (13.4)	5 (17.9)	<0.001*	146 (83.0)	3 (12.0)	2 (40.0)	<0.05*
54–75	120 (30.1)	75 (67.0)	14 (50.0)		30 (17.0)	22 (88.0)	2 (40.0)	
>75	41 (10.3)	22 (19.6)	9 (32.1)		0 (0.0)	0 (0.0)	1 (20.0)	
BMI (kg/m^2^) (mean ± SD)	(31.6 ± 8.5)a	(37.1 ± 11.7)b	(37.2 ± 11.7)b	<0.001*	(31.7 ± 7.7)a	(31.8 ± 4.6)a	(40.7 ± 13.3)b	<0.05*
Smoking status	64 (11.5)	12 (10.9)	1 (3.6)	**>0.05**	19 (10.2)	4 (16.0)	0 (0.0)	**>0.05**
Comorbidity								
CVD	34 (8.5)	44 (39.3)	19 (67.0)	<0.001*	8 (4.3)	6 (24.0)	4 (80.0)	<0.001*
CNS	13 (3.3)	16 (14.3)	3 (10.7)	<0.001*	2 (1.1)	1 (4.0)	0 (0.0)	>0.05
COPD	21 (5.3)	24 (21.4)	9 (32.1)	<0.001*	0 (0.0)	4 (16.0)	0 (0.0)	<0.001*
Malignancy	21 (5.3)	5 (4.5)	1 (3.6)	**>0.05**	4 (2.3)	1 (4.0)	0 (0.0)	**>0.05**
OSA	22 (5.5)	14 (12.5)	10 (35.7)	<0.001*	7 (4.0)	1 (4.0)	2 (40.0)	<0.001*
Asthma	65 (16.3)	16 (14.3)	5 (17.9)	**>0.05**	31 (17.6)	5 (20.0)	1 (20.0)	**>0.05**
HTN	157 (39.3)	83 (74.1)	22 (78.6)	<0.001*	42 (23.9)	17 (68.0)	3 (60.0)	<0.001*
DM	98 (24.6)	53 (47.3)	10 (35.70	<0.001*<0.001*	26 (14.8)	9 (36.0)	1 (20.0)	<0.05*
Dyslipidemia	116 (29.1)	78 (69.6)	15 (53.6)		27 (15.3)	18 (27.0)	2 (40.0)	<0.001*
No of comorbidities Median (interquartile range)	2 (0–7)	3 (0–8)	4 (1–8)	<0.001*	1 (0–7)	3 (0–6)	4 (1–4)	<0.001*
Inpatient admission	223 (55.9)	87 (77.7)	23 (82.1)	<0.001*				
Bleeding events	6 (1.5)	6 (5.4)	2 (7.1)	<0.05*				
Clotting events	19 (4.2)	7 (6.3)	2 (7.1)	**>0.05**				
Death	24 (6.0)	15 (13.4)	6 (21.4)	<0.001*				
Time to study (day) Median (interquartile range)	6a (1–53)	7a (1–42)	12b (1–53)	<0.001*				

*Note*: a,b, the alphabet of different symbols shows statistically significant difference.

Abbreviations: AC, anticoagulation only group; ASA, acetylsalicylic acid; BMI, body mass index; CNS, cerebrovascular disease; COPD, chronic obstructive pulmonary disease; CVD, cardiovascular disease (CAD, CHF, valvular disease, cardiomyopathies); DM, diabetes mellitus; OSA, obstructive sleep apnea; HTN, hypertension; SC, standard care group.

* *p* < 0.05 there is a statistically significant difference.

Quantitatively, before separation into inpatient and outpatient arms, all studied outcomes showed a statistically significant difference between groups (*p* < 0.05) including inpatient admission, bleeding events, and death with the exception of clotting events. Patients in the AC group followed by the ASA group had statistically higher rates of inpatient admission when compared to the SC group (AC = 23 [82.1%], ASA = 87 [77.7%], and SC = 223 [55.9%]), bleeding events (AC = 2 [7.1%], ASA = 6 [5.4%], and SC = 6 [1.5%]), and death (AC = 6 [21.4%], ASA = 15 [13.4%], and SC = 24 [6.0%]) (Table 2).

Of the total study population, 206 (38.2%) were *outpatient* patients and were not admitted to the hospital during the study period. Outpatient cases were distributed and grouped as follows: SC *n* = 176 (85.4%), ASA *n* = 25 (12.1%), and AC *n* = 5 (2.4%). There was a statistically significant difference (*p* < 0.05) between the SC group and other groups regarding the groups: age, number of comorbidities, and comorbidities except for asthma, and malignancy. The SC group was significantly younger and had a lower median number of comorbidities compared to the ASA and AC groups (1 vs. 3 vs. 4, respectively). A large proportion of the outpatient SC group did not have any of the studied comorbidities (*n* = 57 [32.4%]), a statistically significant difference from the AC and ASA groups (*n* = 0 [0%] and *n* = 1 [4%]) (Table 2).

There were 333 patients identified for the COVID‐19 *inpatient* portion of this study. These were distributed into the following: SC *n* = 223 (66.9%), ASA *n* = 87 (26.1%), and AC *n* = 23 (6.9%). There was a statistically significant difference (*p* < 0.05) between the SC group and other groups regarding age, mean number of comorbidities, and comorbidities except for asthma, and malignancy. Again, the SC group was significantly younger with fewer comorbidities than the other groups. All studied primary and secondary outcomes did not show statistically significant differences except for mean length of stay (LOS), which was significantly longer in the AC when compared to SC and ASA (12 vs. 7 vs. 6 days, *p* = 0.00) (Table 3).

**Table 3:** The demographic and clinical characteristics among inpatient group (*n* = 333).

	SC group, no = 223, F (%)	ASA group, no = 87, F (%)	AC group, no = 23, F (%)	*p* Value
Sex				
Female	104 (46.6)	36 (41.4)	9 (39.1)	**>0.05**
Male	119 (53.4)	51 (58.6)	14 (60.9)	
Age (year) mean ± SD	56.9 ± 17.4	66.8 ± 11.8	68.2 ± 15.1	>0.001
Age groups (years)	92 (41.3)	12 (13.9)	3 (13.0)	
<54	90 (40.4)	53 (60.9)	12 (52.2)	
54–75	41 (18.4)	22 (25.3)	8 (34.8)	
>75				
BMI (kg/m^2^)				<0.05*
Mean ± SD	31.6 ± 8.9	30.9 ± 9.0	36.9 ± 11.4	
Smoking	27 (12.1)	8 (9.2)	1 (4.3)	0.71
Comorbidities				
No comorbidity	26 (11.7)	3 (3.4)	0 (0.0)	<0.001*
CVD	26 (11.7)	38 (43.7)	15 (65.2)	<0.001*
CNS	11 (4.9)	15 (17.2)	3 (13.0)	>0.001
COPD	21 (9.4)	20 (23.0)	9 (39.1)	**>0.05**
Malignancy	17 (7.6)	4 (4.6)	1 (4.3)	>0.001
OSA	15 (6.7)	13 (14.9)	8 (34.8)	**>0.05**
Asthma	34 (15.2)	11 (12.6)	4 (17.4)	>0.001
HTN	115 (51.6)	66 (75.9)	19 (82.6)	
DM	72 (32.3)	44 (50.6)	9 (39.1)	**>0.05**
Dyslipidemia	89 (39.9)	60 (69.0)	13 (56.5)	>0.001
LOS (days)				
Median (interquartile range)	6 (1–53)	7 (1–42)	12 (1–53)	**<0.05**
30‐day hospital readmission rate	25 (11.2)	13 (14.9)	5 (21.7)	**>0.05**
ICU admission	77 (34.5)	28 (32.2)	9 (39.1)	**>0.05**
Time needed in ICU before successful discharge (days)	4.5 (1–39)	9 (1–41)	6 (1–20)	**>0.05**
Median (interquartile range)				
Occurrence of ARDS	40 (17.9)	13 (14.9)	5 (21.7)	**>0.05**
Ventilation required	26 (11.7)	13 (14.9)	5 (21.7)	**>0.05**
Study time from admission to intubation (days)	*T* = 12 (3.0)	*T* = 9 (2.3)	*T* = 2(7.1)	>**0.05**
Median (interquartile range)	3.5 (1–24)	2 (1–15)	1	
Time from intubation to successful extubation (days)				**>0.05**
Median (interquartile range)				
F (%)	7 (1–30)	10.5 (2–30)	5.50 (5.50 ± 0.7)	
	8 (3.6%)	5 (5.7%)	1 (3.6%)	
Bleeding events	6 (2.7)	6 (6.9)	2 (8.7)	**>0.05**
Clotting events	18 (8.1)	7 (8.0)	2 (8.7)	**>0.05**
Death	**24 (10.8)**	**15 (17.2)**	**6 (26.1)**	**>0.05**
The outcomes				
No complications	**182 (81.6)**	**64 (73.6)**	**16 (69.0)**	
Clotting	**13 (5.8)**	**5 (5.7)**	**1 (4.3)**	
Clotting + bleeding	**2 (0.9)**	**0**	**0**	
Bleeding	**2 (0.9)**	**2 (2.3)**	**0**	
Death	**20 (9.0)**	**12 (13.8)**	**4 (17.3)**	
Clotting + death	**2 (0.9)**	**2 (2.3)**	**1 (4.4)**	**>0.05**
Bleeding + death	**1 (0.4)**	**1 (1.1)**	**0**	
Clotting + bleeding + death	**1 (0.4)**	**1 (1.1)**	**1 (4.3)**	

Abbreviations: AC, anticoagulation only group; ASA, acetylsalicylic acid; BMI, body mass index; CNS, cerebrovascular disease; COPD, chronic obstructive pulmonary disease; CVD, cardiovascular disease (CAD, CHF, valvular disease, cardiomyopathies); DM, diabetes mellitus; LSA, length of stay; OSA, obstructive sleep apnea; HTN, hypertension; SC, standard care group.

*p < 0.05.

Through multivariate analysis, the odds of inpatient admission were found to be equivalent among all groups when adjusted for age and comorbidity (Table 4). Secondary outcomes were also assessed, including 30‐day readmission rates, the occurrence of ARDS, MICU admissions, and mortality. ASA trended toward reduced OR when compared to SC for all categories but did not reach statistical significance, the most notable being MICU admission (OR 0.63, 95% CI 0.34–1.17, *p* = 0.32). AC trended toward increased OR when compared to SC for almost all categories, but again did not reach statistical significance, most notably 30‐day readmission rates (OR 1.4, 95% CI 0.41–4.8, *p* = 0.58). MICU admission in this group was noted to be at decreased risk (OR 0.82, 95% CI 0.29–2.26) (Table 5).

**Table 4:** Binary logistic regression analysis for predicting primary outcome (inpatient admission).

Variables	Adjusted models		*B*	*p* Value
	OR	95% CI		
Constant	0.54		−0.61	<0.001*
Age groups (year)				
<54	**1**			
54–75	**2.57**	**0.53–1.78**	**0.95**	<**0.001** *
>75	**59.3**	**0.29–3.33**	**4.08**	<**0.001** *
Comorbidity				
CVD	**1.25**	**(0.63–2.44)**	**0.22**	>0**.05**
CNS	**1.92**	**(0.52–7.08)**	**0.65**	>**0.05**
HTN	**1.36**	**(0.85–2.17)**	**0.30**	>**0.05**
DM	**1.78**	**(1.07–2.96)**	**0.58**	<**0.05** *
Dyslipidemia	**1.09**	**1.09 (0.66–1.80)**	**0.08**	>**0.05**
COPD	**3.07**	**(1.01–9.31)**	**1.12**	>**0.05**
OSA	**1.79**	**0.79–4.04)**	**0.59**	>**0.05**
Groups				
Control (SC)	**1**			
ASA	**0.976 (0.53–1.78)**	**0.53–1.78)**	**−0.024**	>**0.05**
AC	**0.989 (0.29–3.33)**	**0.29–3.33)**	**−0.011**	>**0.05**

*Note*: A binary logistic regression model; Hosmer and Lemeshhow test; *χ* ^2^ (12.9), *df* (8), *p *(0.11). Omnibus test model *χ* ^2^ (142.3), *p* (0.00*). Cox and Snell *R* ^2^ = 0.23. Nagelkerke *R* ^2^ = 0.32. Overall percentage (71.1).

Abbreviations: AC, anticoagulation only group; ASA, acetylsalicylic acid; CI, confidence interval; CNS, cerebrovascular disease; COPD, chronic obstructive pulmonary disease; CVD, cardiovascular disease; DM, diabetes mellitus; OSA, obstructive sleep apnea; OR, odds ratio; HTN, hypertension; SC, standard care group.

* *p* < 0.05, statistically significant difference.

**Table 5:** Binary logistic regression analysis for predicting secondary outcomes (30‐day readmission rates, ICU admission, ARDS occurrence, and death among inpatient cases).

	30‐day readmission rate		Occurrence of ARDS		MICU admission		Death	
Variables	Adjusted models OR (95% CI)	***p***	Adjusted models OR (95% CI)	***p***	Adjusted models OR (95% CI)	***p***	Adjusted models OR (95% CI)	***p***
Constant	**0.06**	**<0.001** *	0.13	**<0.001** *	**0.13**	**<0.001** *	**0.014**	**<0.001** *
Age groups (year)								
<54	**1**	>**0.05**	1	>0.05	1	>0.05	1	**<0.05** *
54–75	**1.89 (0.74–4.8)**	>**0.05**	1.26 (0.59–2.66)	>0.05	1.28 (0.70–2.33)	>0.05	4.86 (1.04–22.5)	<0.001*
>75	**0.86 (0.26**–**2.97)**		1.24 (0.49–3.17)		0.91 (0.41–1.96)		10.94 (2.20–45.2)	
Comorbidity								
CVD	**2.6 (1.16**–**5.61)**	<**0.05** *	0.69 (0.31–1.56)	>0.05	0.83 (0.44–1.55)	>0.05	1.24 (0.54–2.83	>0.05
CNS	**1.57 (0.55–4.5)**	>**0.05**	0.62 (0.19–1.94)	>0.05	1.71 (0.74–3.92)	>0.05	1.86 (0.72–4.79	>0.050
HTN	**1.59 (0.69–3.69)**	>**0.05**	1.17 (0.59–2.34)	>0.05	1.40 (0.74–3.92)	>0.05	1.76 (0.71–4.39)	>0.05
DM	**1.32 (0.65–2.69)**	>**0.05**	1.98 (1.06–3.68)	**<0.05** *	1.59 (0.95–2.64)	>0.05	1.25 (0.61–2.58)	>0.05
Dyslipidemia	**0.89 (0.42–1.89)**	>**0.05**	1.15 (0.59–2.21)	>0.05	1.01 (0.59–1.73)	>0.05	0.94 (0.43–2.06)	>0.05
COPD	**0.78 (0.31–2.01)**	>**0.05**	1.25 (0.5–2.66)	>0.050	1.78 (0.91–3.48)	>0.05	3.37 (1.56–7.26)	<0.001*
OSA	**0.50 (0.15–1.65)**	>**0.05**	1.29 (0.52–3.22)	>0.05	1.60 (0.75–3.42)		0.64 (0.19–2.09)	>0.05
Groups								
Control (SC)	**1**		1		**1**		1	
ASA	**0.81 (0.35–1.88)**	>**0.05**	0.71 (0.33–1.52	>0.05	**0.63 (0.34–1.17)**	>0.05	0.85 (00.36–1.99)	>0.05
AC	**1.4 (0.41–4.8)**	>**0.05**	1.19 (0.36–3.99)	>0.05	**0.82 (0.29–2.26)**	>0.05	1.24 (0.37–4.16)	>0.05

*Note*: *ARDS, A binary logistic regression model*; Hosmer and Lemeshhow test; *χ* ^2^ (11.1), *df* (7), *p* (0.13). Omnibus test model *χ* ^2^ (10.09) *p* (0.52). Cox and Snell *R* ^2^ = 0.03. Nagelkerke *R* ^2^ = 0.05. Overall percentage (82.6%) ICU.

*ICU, A binary logistic regression model;* Hosmer and Lemeshhow test; *χ* ^2^ (2.18), *df* (7), *p *(0.97). Omnibus test model *χ* ^2^ (10.09) *p* (0.52). Cox and Snell *R* ^2^ = −0.05. Nagelkerke *R* ^2^ = 0.08. Overall percentage = 67.3%.

Abbreviations: AC, anticoagulation only group; ASA, acetylsalicylic acid; CI, confidence interval; CNS, cerebrovascular disease; COPD, chronic obstructive pulmonary disease; CVD, cardiovascular disease; DM, diabetes mellitus; OSA, obstructive sleep apnea; OR, odds ratio; HTN, hypertension; SC, standard care group.

* *p* < 0.05, statistically significant difference.

## 4 DISCUSSION

This record‐based analytical cross‐sectional study reported that the use of low‐dose ASA has no statistical significance but a potential protective role in the management of COVID‐19 as it was associated with a lower risk of 30‐day hospital readmission, ICU admission, need for mechanical ventilation, ARDS, and mortality when compared to the SC. It showed that AC increases the risk of all studied outcomes except the risk of ICU admission, which was decreased, compared to the SC. However, multicenter randomized control trials are needed to assess the causality effects of these therapies.

In this study, the randomly selected patients were distributed into the three management protocols, in which the majority of COVID‐19 cases were managed with the SC protocol (74%), and the remaining were managed with prophylactic AC and ASA (20.8%), and therapeutic anticoagulation (5.2%). Due to this distribution, more than 80% of COVID‐19 cases had mild symptoms, with critical cases being less than 10%.^12^ There was a statistically significant difference (*p* < 0.05) between the SC group and other groups regarding age, mean number of comorbidities, and all individual comorbidities except for asthma, and malignancy. This finding is related to the indication for ASA and therapeutic AC use in the ASA and AC groups, respectively. In the studied population, ASA was primarily used for secondary prevention of thromboembolic diseases such as coronary artery disease, cerebrovascular disease, and peripheral vascular disease. As a result, the ASA and AC groups contained patients with a much higher number of comorbidities, than the SC group. Patients on ASA tend to have more risk factors for severe COVID‐19 infection (e.g., older age, CAD, DM, HTN, CVD, COPD, etc.) and thus generally have worse outcomes.

The duration of hospital stay (days) was significantly higher in the AC group (median 12 days) compared to other groups. Moreover, the odds of secondary outcomes, including 30‐day readmission, the occurrence of ARDS, ICU admission, and mortality were also higher in the AC group. This result is in agreement with recent literature, most notably a recent study by Sadeghipour et al., who concluded that routine use of intermediate‐dose anticoagulation did not decrease the severity of COVID‐19 and had no mortality benefit.^13^

ASA reduced 30‐day readmission rates, the occurrence of ARDS, ICU admissions, and mortality. We hypothesize that this effect is due to ASA's anti‐inflammatory effect, antiplatelet aggregation, pleiotropic effects, and potential antiviral effect.^8^ A study by Chow et al. showed that patients using ASA had less severe illness, required less oxygen support on admission, and overall had a more favorable outcome.^11^ Merzon et al. found that ASA significantly decreases the disease duration time between first positive and first negative tests (19.8 ± 7.8 vs. 21.9 ± 7.9 days, *p* = 0.045), further supporting the role of ASA in COVID‐19.^14^

Among the inpatient COVID‐19 studied cases, there was no statistically significant difference regarding clinical outcomes between the studied groups, which is similar to results reported by Chow et al. and Yuan et al.^11^, ^15^ Multivariate analysis, however, showed that ASA slightly reduces inpatient admission when compared to the SC and AC groups (OR 0.976 CI [0.53–1.78]) and (OR 0.989 CI [0.29–3.33]), respectively, indicating that ASA may have a slight protective role in preventing inpatient admissions. This result is similar to a cohort study done in Iran by Haji Aghajani et al., which showed a higher rate of mortality in hospitalized patients but, when adjusted for comorbidities, also showed a protective effect of ASA.^16^

Low‐dose ASA was shown to reduce the risk of ARDS (OR 0.71, 95% CI 0.33–1.52, *p* = 0.38) when compared to SC. This is in agreement with Zhou et al. who proposed that low‐dose ASA administration before hospitalization can prevent severe ARDS in COVID‐19 and decrease the risk of serious complications.^17^ ASA and AC both reduced the risk of ICU admission ([OR 0.63, 95% CI (0.34–1.17]) and (OR 0.82, CI [0.29–2.26]), which is consistent with Chow et al.^11^ This can be explained by the effective role of Low‐dose ASA in reducing the risk of ARDS and mechanical ventilation.^11^

Low‐dose ASA decreased the risk of mortality among COVID‐19 cases in the multivariate analysis (OR 0.85, 95% CI 0.36–1.99, *p* = 0.71) when compared to the SC group. Our data are similar to other studies that found a nonsignificant reduction in mortality.^6^, ^15^ In contrast, there was no association between ASA and mortality in a meta‐analysis performed by Salah et al., Formiga et al., and a single‐center retrospective study in China.^7^, ^18^, ^19^ Differences in setting, general condition of patients, time of diagnosis, SC, and study design can explain the differences between our studies and these.

This record‐based analytical cross‐sectional study reported that the use of low‐dose ASA has no statistical significance but a potential protective role in the management of COVID‐19 as it was associated with a lower risk of 30‐day hospital readmission, ICU admission, need for mechanical ventilation, ARDS, and mortality when compared to the SC. It showed that AC increases the risk of all studied outcomes except the risk of ICU admission, which was decreased, compared to the SC. Low‐dose ASA is a low‐cost, widely available, pleiotropic medication used for primary and secondary prevention of multi‐system diseases and pathologies.^8^ Multicenter randomized control trials are needed to assess the causality effects of these therapies.^20^

*The main limitations of* our study are that it was a single‐center, record‐based, retrospective study, and that the AC group was small in number. The study design is a descriptive cross‐sectional study. It is classified by the proportion of the use of each, which describes the prevalence of use of each (what's going on at Upstate University).

*The main strengths of our study* were that the relatively large sample size was collected over the long duration of the pandemic, from March until December, and the many outcomes were studied in detail. Every case fulfilling the selection criteria has an equal chance of being recruited into the sample (so it is a random sample) to find the association and risk assessment.

## 5 CONCLUSIONS

Low‐dose ASA is a promising, effective, protective medication in potentially improving the outcomes in COVID‐19 cases, through reducing mortality, and morbidity (hospital readmission rates, the occurrence of ARDS, and ICU admission) but not statistically significant due to the higher risk patients in these groups. further prospective research is still needed.

## 6 RECOMMENDATION

Further multicenter, prospective, randomized controlled trials with sufficient matching are required to further investigate the role and mechanism of ASA in the management of COVID‐19. We encourage further investigation of easily accessible, common, and safe medications with notable mechanisms of action to aid in the fight against COVID‐19‐related morbidity and mortality.

## AUTHOR CONTRIBUTIONS

**Muhammad B. Malik**: Conceptualization; investigation; methodology; project administration; resources; validation; writing—original draft; writing—review and editing. **Samar A. Amer**: Conceptualization; formal analysis; visualization; writing—original draft; writing—review and editing. **Eric Merrell**: Conceptualization; investigation; writing—original draft; writing—review and editing. **Ronald Russo**: Conceptualization. **Jeffrey B. Riley**: Formal analysis; methodology; resources; validation. **Elizabeth James**: Investigation. **Anderson Anuforo**: Investigation. **Soumya Adhikari**: Investigation. **Philip Chebaya**: Investigation. **Edward Darling**: Writing—original draft. **Michael Kuhn**: Writing—original draft. **Gary Nieman**: Writing—original draft. **Ahmed Shawkat**: Conceptualization. **Hani Aiash**: Conceptualization; methodology; project administration; resources; supervision; validation; writing—review and editing. All authors have read and approved the final version of the manuscript.

## CONFLICT OF INTEREST

The authors declare no conflict of interest.

## TRANSPARENCY STATEMENT

I affirm that this manuscript is an honest, accurate, and transparent account of the study being reported; that no important aspects of the study have been omitted; and that any discrepancies from the study as planned (and, if relevant, registered) have been explained.

## ETHICS STATEMENT

This study was reviewed by the institutional review board (IRB) at Upstate Medical University and was considered exempt according to the #4(iii) exemption category in federal regulations. The requirement for written informed consent was waived by the IRB and the study was conducted in accordance with ethical principles.

## Data Availability

The datasets used and/or analyzed during the current study are available from the corresponding author on reasonable request or email dr_samar11@yahoo.com.
